# Cost-Effectiveness and Budget Impact Analysis of the Use of Faricimab in Diabetic Macular Edema and Neovascular Age-Related Macular Degeneration in Colombia

**DOI:** 10.36469/001c.129832

**Published:** 2025-03-04

**Authors:** Daniel Samacá-Samacá, Antonio Robles, Hugo Ocampo, Francisco J. Rodríguez, Carolina Sardi-Correa, Laura Prieto-Pinto, Christian Bührer, Camilo Tamayo, David Rodríguez, Mauricio Hernández-Quintana

**Affiliations:** 1 Evidence Generation, Roche Colombia, Bogotá; 2 Clínica de Oftalmología, Cali, Colombia; 3 Fundonal; 4 Escuela de Medicina y Ciencias de la Salud, Universidad del Rosario, Bogotá, Colombia; 5 Instituto Nacional de Investigación en Oftalmología, Medellín, Colombia; 6 Hoffman-La Roche, Basel, Switzerland; 7 IQVIA Colombia, Bogotá; 8 Departamento Médico, Roche Colombia, Bogotá

**Keywords:** cost-effectiveness analysis, budget impact analysis, macular degenerations, diabetic macular edema, Colombia

## Abstract

**Background:** Retinal diseases are major contributors to disability, significantly affecting patients’ quality of life. Diabetic macular edema (DME) and neovascular age-related macular degeneration (nAMD) represent a significant disease and economic burden in Colombia. Assessing the economic evaluation of available treatments is essential for the efficient allocation of healthcare resources. **Objective:** To estimate the cost-effectiveness and budget impact of using faricimab for the treatment of patients with DME and nAMD within the Colombian health system. **Methods:** This study used a 25-year Markov cohort model to estimate the cost-effectiveness of faricimab vs aflibercept, ranibizumab, and brolucizumab. We used the methodological guidelines of the local health technology assessment agency for conducting the cost-effectiveness and budget impact analysis. Transition probabilities and injection frequencies were derived from the literature. Drug prices were retrieved from public local databases. Quality-adjusted life years (QALYs) were assessed. The potential patient population for the budget impact analysis was estimated based on disease prevalence and expert consultations. **Results:** Faricimab treat-and-extend (T&E) was dominant vs aflibercept T&E (+0.22 QALYs), ranibizumab T&E (+0.55 QALYs), and brolucizumab for 8 to 12 weeks (+0.06 QALYs) in DME, generating cost savings (in US dollars) of 3849,1375, and 2824,respectively.InnAMD,faricimabalsoshoweddominancevsafliberceptasneeded(+0.12QALYs),ranibizumabasneeded(+0.05QALYs),andbrolucizumab8to12weeks(+0.12QALYs)withsavingsin(US)7223, 5792,and6798, respectively. Assuming an annual market share increase for faricimab of 15% for DME and 13% for nAMD, the Colombian Health System could save 144millionover3years.Ofthesesavings,122.7 million are attributed to drug costs and 21.3milliontoadministrationcosts(US1 = Col$4325). **Conclusion:** Considering a willingness to pay threshold of $5988 per additional QALY, faricimab is a cost-effective alternative for both DME and nAMD for the Colombian healthcare system, showing dominance over other anti–vascular endothelial growth factor agents. Faricimab provides better health outcomes at lower costs vs other treatments.

## BACKGROUND

Blindness and vision loss are major contributors to disability, impacting individuals’ quality of life and often leading to diminished financial, educational, and employment opportunities.^1^ According to the Global Burden of Disease Study, in 2020, 7.8 million people in Colombia had vision loss, of whom 300 000 had blindness.^2^

Diabetic macular edema (DME) is the primary cause of blindness, affecting 1 in 15 patients with diabetes.^3,4^ In cases of diabetic retinopathy, 2.8% experience moderate to severe vision loss and 6.8% suffer from blindness, with DME accounting for 50% of these cases.^5^ The prevalence of DME has been estimated at 5.81% in low- and middle-income countries and 5.14% in high-income countries.^6^

Although neovascular age-related macular degeneration (nAMD) represents 1% to 15% of the total cases of age-related macular degeneration (AMD), it accounts for more than 80% of cases of severe visual loss or blindness,^7^ leading to a considerable impact on patient well-being.^8^

In 2021, the disease burden from visual impairment in Colombia was estimated at 258 594 disability-adjusted life years (DALYs).^9^ Local estimates indicate there were 24 947 cases of DME with moderate-to-severe vision loss and blindness, corresponding to 5229 DALYs.^9^ There were 15 783 cases of visual impairment attributed to nAMD, associated with 3055 DALYs, highlighting the significant disease burden in the Colombian population.^9^

Furthermore, the total economic burden associated with DME and nAMD was approximately $834.9 million and $193.9 million (US dollars), respectively, with direct costs representing 89% to 93% of the total expenses in 2022.^10^ Indirect costs related to both diseases were estimated at $93.3 million and $13.9 million for DME and nAMD, respectively.^10^ These indirect costs included loss of access to work, absenteeism, transportation costs, and the caregiver’s loss of productivity.^10^ The burden related to indirect costs reflects the effect that diseases have on the burden of patients and caregivers, highlighting the need to prioritize effective interventions that reduce the burden of disease while contributing to the financial sustainability of the healthcare system.

Both DME and nAMD are treated with anti–vascular endothelial growth factor (anti-VEGF), including aflibercept, ranibizumab and brolucizumab.^11–13^ Recently, faricimab, a new anti-VEGF, has been approved in Colombia. Faricimab targets and inhibits Ang-2 and VEGF-A, a mechanism of action suggested as an advantage in controlling the disease.^14^ Recent studies have shown that faricimab reduces the number of injections required for treating DME and nAMD, indicating a reduction in the burden of treatment and possibly in the cost of managing both diseases.^15,16^

The objective of this study was to estimate the cost-effectiveness and budget impact of using faricimab, compared with other anti-VEGF agents approved in Colombia, for the treatment of patients with DME and nAMD.

## METHODS

### Cost-Effectiveness Analysis

A local adaptation of a previously implemented faricimab cost-effectiveness model was conducted,^17^ following the guidelines of the local health technology assessment agency (Instituto de Evaluación Tecnológica en Salud [IETS]).^18^ The cost-effectiveness threshold used was 86% of GDP per capita, as estimated for Colombia by Espinosa et al, corresponding to US $5988 for 2023 (US $1 = Col$4325 COP).^19^

### Effectiveness Inputs

The efficacy of faricimab in DME was evaluated in 2 phase 3 double-blind randomized clinical trials, YOSEMITE and RHINE, that assessed the noninferiority of faricimab in extended doses compared with a fixed 8-weekly aflibercept dose.^20^ The results of these studies showed that faricimab treat and extend (T&E) had noninferior visual acuity gains compared with aflibercept, with fewer injections required.^20^ Similarly, for nAMD, TENAYA and LUCERNE, 2 noninferiority phase 3 double-blind randomized clinical trials, demonstrated that the change in best-corrected visual acuity (BCVA) of faricimab T&E was noninferior to aflibercept every 8 weeks.^21^

However, for the economic model, the transition probabilities between VA states were estimated from network meta-analyses (NMA) comparing faricimab T&E with other flexible regimens (T&E or as needed), as head-to-head studies only compared faricimab fixed-dose regimens.

For DME, an NMA of faricimab T&E compared with ranibizumab and aflibercept, on an as-needed schedule, and brolucizumab, every 8 to 12 weeks, was used.^15^ The NMA results showed that faricimab T&E achieved greater or equal gains in BCVA compared with other anti-VEGF agents.^15^

The same NMA provided the difference in the number of injections between faricimab T&E, compared with aflibercept as needed and ranibizumab as needed, for the first year of treatment while the difference between faricimab T&E and brolucizumab 8 to 12 weeks was estimated based on the results of the Wykoff et al study.^22^ The number of injections for subsequent years was estimated based on the results from the Protocol T study andWykoff et al.^22,23^

For nAMD, faricimab T&E was compared with ranibizumab T&E, aflibercept T&E, and brolucizumab every 8 to 12 weeks. A recent NMA for nAMD found that faricimab T&E showed comparable efficacy vs other extended regimens while reducing the number of injections required.^16^ This NMA also provided the annual difference in the number of injections between faricimab T&E vs aflibercept T&E and ranibizumab T&E.^16^ The difference in the number of injections compared with brolucizumab every 8 to 12 weeks was not estimated in the NMA; therefore, it was estimated based on the results of Finger et al.^24^

### Cost Inputs

National public databases were used to estimate the acquisition cost of ranibizumab, aflibercept, and brolucizumab. The weighted average price for each drug presentation (prefilled syringe or vial) was obtained from the drug pricing information system (Sistema de Información de Precios de Medicamentos [SISMED]) for 2023. The price of faricimab was provided by the manufacturer.

The frequency of healthcare resources used in the treatment of DME and nAMD was validated with clinical experts to define a representative case. Costs of these healthcare resources were established using national public databases. We used a healthcare payer perspective, which included direct medical costs: drug acquisition costs, administration costs, visual impairment and blindness costs (supportive care), and management of adverse events.

Input parameters for administration, supportive care, and adverse event costs are presented in **Supplementary Table S1**. Administration costs included the operating room for intravitreal injection and the costs associated with supplies and health personnel. It also included coherent optical tomography, which is the standard imaging to assess the need for treatment. Supportive care costs included the vision-related resources needed when a patient’s visual acuity (VA) reaches a threshold level of impairment.^25^ This included rehabilitation for low vision, low-vision aids, services to treat vision loss–related depression, and hip prostheses due to falls.^25^

Based on the information from the NMAs, no differences between anti-VEGFs in the occurrence of adverse events were identified.^15,16^ Therefore, the model included the costs of adverse events treatment assuming the same probability of occurrence among all treatments. Prices for administration, supportive care, and adverse events were obtained from the Integrated Social Protection Information System.

### Markov Cohort Model

**Figure 1.**
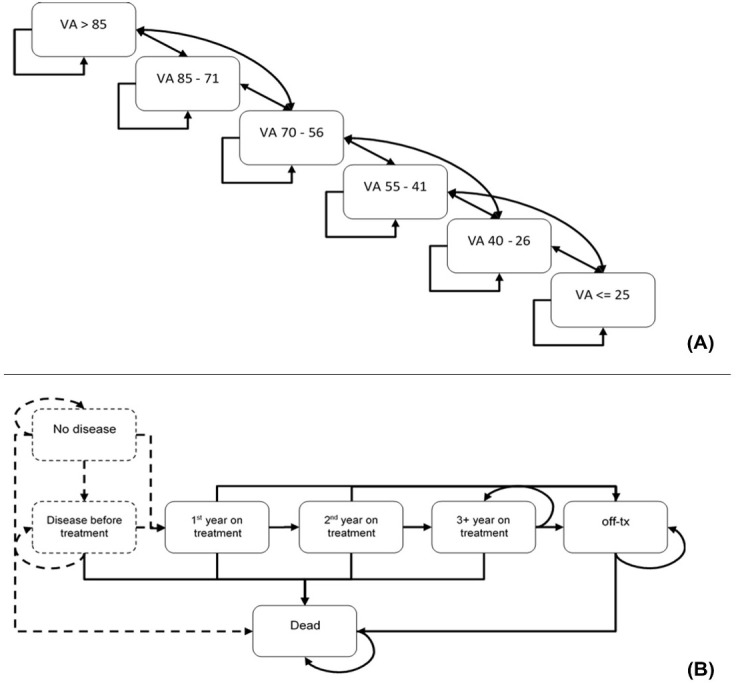
Structure of the Markov Model (A) and Treatment Pathway between Health State Categories (B) Abbreviation: VA, visual acuity.

A Markov cohort model (**Figure 1**) was used to estimate changes in bilateral VA, annual injection frequency, and associated costs for the Colombian Health System. The time horizon was 25 years, considering the age at disease onset and life expectancy. The outcome assessed was quality-adjusted life years (QALYs). The duration of the cycles was 4 weeks, which provided greater flexibility and precision in calculating doses and treatment costs. An annual discount rate of 5% was applied to costs and health outcomes, following the guidelines of the IETS.^18^ The Markov model assumed a treatment duration of 5 years. After 5 years, it was assumed that 85% of patients discontinued treatment.^23^ The details of the model parameters are presented in **Table 1**.

**Table 1.** Cost-Effectiveness Model and Budget Impact Analysis Inputs

**Variable**	**Value**	**Source**
Cost-effectiveness model (base case)		
Time horizon (based on disease age at onset)	25 years	National Institutes of Health^26–28^
Annual discount (costs and effects)	5%	IETS^18^
Treatment discontinuation after 5 years	85%	Glassman et al^23^
Distribution of VA	(See **Table S2**)	NICE,^29^ Wykoff et al^30^
Utility values	(See **Table S3**)	Czoski-Murray et al^31^
Disutility of intravitreal injection	50%	NICE^29^
Baseline incidence of DME in 2nd eye	46.5%	NICE^32^
Monthly incidence of DME in 2nd eye	0.8%	NICE^32^
Baseline incidence of nAMD in 2nd eye	7.3%	NICE^32^
Monthly incidence of nAMD in 2nd eye	1.4%	NICE^32^
Budget impact analysis		
Diabetes prevalence	8.4%	IDF Diabetes Atlas^33^
DME prevalence among diabetic patients	4.92%	Teo et al^34^
% diagnosed patients (DME)	85%	Assumption based on clinical experts
% DME patients treated with anti-VEGF agents	100%	Assumption based on clinical experts
Prevalent cases with bilateral disease (DME in both eyes)	65%	Assumption based on clinical experts
Annual market share increase (DME)	15%	Assumption based on clinical experts
AMD prevalence	0.32%	Wong et al^35^
Late AMD with neovascular type	51.1%	Wong et al^35^
Prevalent cases with bilateral disease (nAMD in both eyes)	29.2%	Assumption based on clinical experts
% patients treated with anti-VEGF agents	80%	Assumption based on clinical experts
Annual market share increase (nAMD)	13%	Assumption based on clinical experts
Patients with healthcare coverage	98.5%	MinSalud^36^

Abbreviations: AMD, age-related macular degeneration; DME, diabetic macular edema; IETS, Instituto de Evaluación Tecnológica en Salud; nAMD, neovascular age-related macular degeneration; NICE, National Institute for Health and Care Excellence; VA, visual acuity; VEGF, vascular endothelial growth factor.

A detailed description of the model can be found in Bührer et al.^17^ The model was designed to illustrate the natural progression of the disease and the development of both conditions.^17^ The model comprised 6 categories of vision-related health states defined by VA scores: >85, 85-71, 70-56, 55-41, 40-26, and ≤25. A lower score indicates poorer vision (**Figure 1**). These scores reflected the number of letters accurately identified in a VA test, using the ETDRS (Early Treatment Diabetic Retinopathy Study) scale. Patients were initially distributed in the model according to the baseline VA of their first affected eye (**Supplementary Table S2**).^29,30^ Bilateral disease incidence was used to determine which patients were modeled for DME or nAMD in the second eye.^29,32^ Baseline VA in the second eye was used for health state model distribution (**Supplementary Table S2**).^29,30^

The model distinguished 3 phases based on the typical clinical progression of the diseases: Year 1 was the loading phase during which most vision improvements occurred, Year 2 simulated the stabilization of the disease and the maintenance of previously gained vision, and Year 3 and onward involved reduced treatment intensity and long-term maintenance.^17^ During Year 1, patients either remained stable or transitioned between health states, experiencing either improvements or declines in VA. They could advance by up to 2 health states (eg, from VA 55-41 to VA 85-71) or drop by 1 health state. During Year 2, patients could move up or down 1 health state, while in Year 3, patients could move down 1 or 2 health states, and in both years they could remain stable. Utility values and non-treatment-related costs were assigned according to the VA health state category (**Supplementary Table S3**).^17^ A disutility value was applied for every administration of intravitreal injection (**Table 1**).^29^

The transition probabilities were estimated using a multistate model in R programming language, described in Bührer et al.^17^ The transition probabilities represent the changes among VA health states, defined by either a 1- or 2-state increase in VA, a 1- or 2-state decrease, or no change.^17^ A gain of more than 22.5 letters indicates an increase in 2 VA states. A gain between 7.5 and 22.5 letters indicates an increase in 1 VA state. A gain or loss of up to 7.5 letters indicates that the patient remains stable. A loss between 7.5 and 22.5 letters indicates a decrease in 1 state. Finally, a loss of more than 22.5 letters indicates a decrease in 2 states.

During Year 1, transition probabilities are estimated per 4-week cycle, according to the baseline VA distribution and transitioning based on VA gains.^17^ After the first year, transitions become independent of the baseline VA.^17^ Transition probabilities between health states for Years 1, 2, and 3 and onward are presented in **Supplementary Tables S4-S9**.

Subsequently, the VA health states were integrated with the treatment pathway states to assign treatment costs (including acquisition, administration, and monitoring), health state utilities, and treatment-related disutility values (**Figure 1**).

Utility values were obtained from Czoski-Murray et al, in which the relationship between VA scores and utility values was estimated using a regression model with time trade-off methods and incorporating an age covariate.^31^ Utility values according VA scores are presented in **Supplementary Table S3**.

### Sensitivity Analyses

Deterministic and probabilistic sensitivity analyses (PSA) were conducted to assess the uncertainty and explore the robustness of the model results. The deterministic sensitivity analysis was based on the upper and lower limits of the 95% confidence interval of the model parameters, when available, or the 30th and 70th percentile of the point estimate. Parameters included drug acquisition costs, discount rates, cost of administration, year of disease onset, and costs associated with blindness and adverse events management. For acquisition drug costs, an increase and a discount of 10% was used as the upper and lower limits. Net monetary benefit was used as the output for the deterministic sensibility analysis.

For the PSA, 1000 Monte Carlo chain simulations were performed based on the observed probabilistic distribution assigned to each parameter. Beta distributions were used for the proportion of patients requiring visual impairment and blindness aids and for the administration and follow-up visits assigned to the second eye if both eyes were treated. Beta or gamma distributions were used for adverse events management. A gamma distribution was used for drug costs, and a normal distribution was applied for the rest of the parameters. Parameters for PSA are presented in **Supplementary Tables S10 and S11**.

### Budget Impact Analysis

The budget impact analysis (BIA) was conducted according to the guidelines of the local health technology assessment agency.^37^ Bevacizumab (off-label) (for DME and nAMD) and dexamethasone (for DME) were included in the analysis, according to the country’s clinical practice, as validated by clinical experts. The BIA represents the comparison of 2 scenarios: the current scenario without faricimab and a new scenario considering the entry of faricimab onto the market.^37^

To identify the target population, we assessed the total number of individuals at risk of developing the health conditions and who could potentially benefit from the analyzed technologies. The sources for estimating the target population for each disease are presented in **Table 1**.

For AMD, a prevalence rate of 0.32% was applied for individuals over 40 years of age, with 51.1% of late AMD cases classified as neovascular.^35^ This estimate was refined for adults over 50 years. According to the consultation with clinical experts, 29.2% of prevalent cases of nAMD have the disease in both eyes (bilateral disease), and 80% of patients receive anti-VEGF treatment. For the DME estimate, we considered the adult population over 18 years and applied a diabetes prevalence of 8.4%,^33^ with a DME prevalence of 4.92% among patients with diabetes.^34^ According to the clinical experts, 65% of DME-prevalent cases involve bilateral disease and all patients were assumed to be receiving treatment. Health coverage was set at 98.5%.^36^

Subsequently, the current market distribution for the technologies used in managing both diseases and the estimation of how this market distribution would change with the use of faricimab was applied. An annual increase in market share of 15% in DME and 13% in nAMD was assumed for faricimab, based on the clinicians’ expertise.

The costs associated with managing each disease were estimated for each scenario. The cost estimation for drugs and the use of health resources followed the methodology previously described for the cost-effectiveness analysis.

A 3-year time horizon was established for the BIA, with 2024 as the initial year (ie, the analysis extends to 2027). The results are presented for each year and the 3-year cumulative analysis.

## RESULTS

### Cost-Effectiveness Analysis

The comparison between faricimab and other anti-VEGFs included in the analysis during the 25-year horizon is presented in **Table 2**. The results showed that faricimab generates additional QALYs against aflibercept, ranibizumab, and brolucizumab in both DME and nAMD. Additionally, faricimab was associated with lower costs than aflibercept, ranibizumab, and brolucizumab during the analysis period, indicating that faricimab is a dominant alternative to all comparators.

**Table 2.** Cost-Effectiveness Results of Faricimab T&E vs Other Anti-VEGF Agents in Diabetic Macular Edema and Neovascular Age-Related Macular Degeneration

**Treatment**	**Cost (US $)**	**Incremental Cost vs Faricimab**	**QALYs**	**Incremental QALYs vs Faricimab**	**ICER vs Faricimab**	**Faricimab NMB vs Comparator**
Diabetic macular edema (base case)						
Faricimab T&E	17 857	–	7.94	–	–	–
Aflibercept PRN	21 706	3849	7.73	-0.22	Faricimab dominant	5149
Ranibizumab PRN	19 232	1375	7.39	-0.55	Faricimab dominant	4680
Brolucizumab 8-12 weeks	20 681	2824	7.89	-0.06	Faricimab dominant	3163
Diabetic macular edema (PSA)						
Faricimab T&E	21 898	–	7.92	–	–	–
Aflibercept PRN	23 401	1503	7.77	-0.14	Faricimab dominant	2397
Ranibizumab PRN	19 544	-2354	7.57	-0.35	US $11 904	-243
Brolucizumab 8-12 weeks	25 186	3288	7.53	-0.395	Faricimab is dominant	5604
Neovascular age-related macular degeneration (base case)						
Faricimab T&E	28 429	–	5.73	–	–	–
Aflibercept T&E	35 652	7223	5.61	-0.12	Faricimab dominant	7946
Ranibizumab T&E	34 221	5792	5.68	-0.05	Faricimab dominant	6087
Brolucizumab 8-12 weeks	35 227	6798	5.61	-0.12	Faricimab dominant	7540
Neovascular age-related macular degeneration (PSA)						
Faricimab T&E	29 528	–	5.75	–	–	–
Aflibercept T&E	30 839	1311	5.71	-0.03	Faricimab dominant	1521
Ranibizumab T&E	35 662	6134	5.72	-0.03	Faricimab dominant	6294
Brolucizumab 8-12 weeks	37 771	8242	5.71	-0.03	Faricimab dominant	8464

Abbreviations: ICER, incremental cost-effectiveness ratio; NMB, net monetary benefit; PRN, as needed; PSA, probabilistic sensitivity analysis; QALY, quality-adjusted life year; T&E, treat and extend; VEGF, vascular endothelial growth factor.

Note: US $1 = Col$4325.

The detail of the cost components and the reduction in the number of injections is presented in **Table 3**. Drug costs represent the highest component of total costs, followed by costs related to drug administration. The treatment frequency results showed that faricimab requires fewer injections than aflibercept, ranibizumab, and brolucizumab for both diseases, reducing the drug acquisition and administration cost.

**Table 3.** Components of the Analysis of Faricimab T&E vs Other Anti-VEGF Agents in Diabetic Macular Edema and Neovascular Age-Related Macular Degeneration

**Treatment**	**Drug Acquisition Costs (US $)**	**Administration Costs (US $)**	**AE Costs (US $)**	**Cost of Visual Impairment Aids (US $)**	**No. of Injections vs Faricimab**
Diabetic macular edema					
Faricimab T&E	14 039	2363	42	447	–
Aflibercept PRN	16 308	3163	42	486	-10.22
Ranibizumab PRN	13 821	3177	42	559	-10.22
Brolucizumab 8-12 weeks	15 382	2667	42	455	-4.30
Neovascular age-related macular degeneration					
Faricimab T&E	23 956	4032	34	407	–
Aflibercept T&E	29 475	5717	34	426	-24
Ranibizumab T&E	27 464	6313	34	410	-32
Brolucizumab 8-12 weeks	29 628	5137	34	428	-16

Abbreviations: AE, adverse effects; ICER, incremental cost-effectiveness ratio; PRN, as needed; QALY, quality-adjusted life year; T&E, treat and extend; VEGF, vascular endothelial growth factor.

Note: US $1 = Col$4325.

Visual impairment aid costs were similar among all alternatives for both diseases. Due to the lack of reported differences in the risk of adverse events in the clinical trials, validated by clinical experts, the cost of managing adverse events was the same for all technologies for both health conditions.

The results of the deterministic sensitivity analysis showed that the drug acquisition cost for the comparators was the most sensitive parameter in the model, followed in most cases by the faricimab acquisition cost and the discount rate. The incremental net monetary benefit (NMB) of faricimab was positive in all analyses. The tornado diagrams for the deterministic analysis are found in **Supplementary Figures S1-S6**.

**Figure 2.**
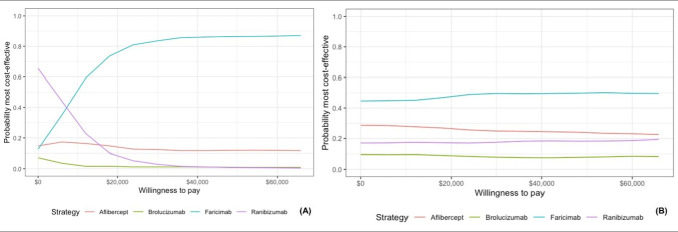
Cost-Effectiveness Acceptability Curve for Diabetic Macular Edema (**A**) and Neovascular Age-related Macular Degeneration (**B**)

The cost-effectiveness acceptability curves are presented in **Figure 2**. The cost-effectiveness acceptability curve indicates the probability of each intervention of being the most cost-effective alternative. The PSA showed that faricimab is likely to be a dominant alternative compared with other anti-VEGF agents in nAMD. At a willingness-to-pay threshold of US $5988, the probability that faricimab of being dominant is 44.8%. In DME, faricimab is likely to be dominant against aflibercept and brolucizumab, with an ICER of $11 904 compared with ranibizumab. The probability of faricimab of being cost-effective with the current threshold is 35%. **Table 2** presents the results of the PSA.

### Budget Impact Analysis

The potential for patients with DME was estimated at 134 094 cases; for nAMD, it was estimated at 24 276 cases in 2024.

**Table 4** presents the results of the BIA. Assuming an annual increase in market share of 15% in DME and 13% in nAMD, the introduction of faricimab would generate savings of US $144 056 764 for the Colombian Health System over 3 years. Of the estimated savings, 86% and 76% correspond to drug acquisition cost savings for DME and nAMD, respectively.

**Table 4.** Budget Impact Analysis of Faricimab in Diabetic Macular Edema and Neovascular Age-Related Macular Degeneration

	**2024 (Base Year) (US $)**	**2025 (US $)**	**2026 (US $)**	**2027 (US $)**	**3-Year Total Budget Impact (US $)**
Diabetic macular edema					
Without faricimab	676 698 039	685 540 737	693 715 771	701 522 835	2 080 779 343
With faricimab	676 698 039	664 704 905	651 547 174	637 558 092	1 953 810 171
Budget impact	–	-20 835 833	-42 168 597	-63 964 743	-126 969 172
Neovascular age-related macular degeneration					
Without faricimab	110 441 179	113 484 762	116 425 426	119 293 199	349 203 387
With faricimab	110 441 179	110 731 085	110 775 363	110 609 347	332 115 795
Budget impact	–	-2 753 677	-5 650 063	-8 683 852	-17 087 592

Note: US $1 = Col$4325.

## DISCUSSION

Cost-effectiveness and budget impact analyses are crucial for enabling policymakers to allocate limited resources more efficiently to interventions that improve health outcomes.^38,39^ This is particularly relevant in countries like Colombia, where challenges in resource management within the health system limit the population’s access to health services.^40^

This study evaluated the potential economic benefit of using faricimab in two visual diseases reported to have a high disease and economic burden that impacts both the healthcare system and the well-being and quality of life of patients and their caregivers.^9,10^ The clinical evidence between faricimab and other anti-VEGFs suggests comparable or higher efficacy in BCVA outcomes^15,16^; therefore, a cost-effectiveness model was conducted to quantify the additional clinical benefit of the new intervention and its relationship with the costs. For the cost-effectiveness model, we used the QALYs metric, which considers an adjustment for the quality of life to reflect the overall impact of the intervention on the patient’s health, allowing differentiation of interventions that might provide the same expected effectiveness outcome but also bestow an additional benefit on a patient’s quality of life.^41^

Our results demonstrated that faricimab is a dominant alternative to other anti-VEGF agents for the treatment of both DME and nAMD. Faricimab achieves savings for the healthcare system while gaining QALYs, thereby generating better health outcomes at a lower cost. The dominance of faricimab over alternatives was maintained in the sensitivity analyses for all comparators in nAMD and against aflibercept and brolucizumab in DME, supporting the robustness of the results. Additionally, over a 3-year horizon, the impact of faricimab adoption would represent savings to the healthcare system of more than US $144 million for both diseases.

The model adapted for this study has been previously used in other countries.^17,42–45^ In Italy^42^ and Peru,^43^ the model was used to estimate the cost-effectiveness of faricimab for DME and nAMD. Additionally, in Canada,^44^ Russia,^45^ and the United Kingdom,^17^ it was adapted for DME. In all studies, faricimab has been found to be a cost-effective alternative associated with a gain in QALYs at a lower cost to the healthcare system.^17,42–45^

The potential savings related to the introduction of faricimab and its dominance over alternative treatments were obtained due to its efficacy and reduced number of injections. A network meta-analysis by Watkins et al found that faricimab was associated with an improvement in the change in BCVA compared with ranibizumab, in an as-needed regimen, while showing a comparable efficacy to aflibercept as-needed treatment.^15^ In nAMD, faricimab has demonstrated comparable efficacy to other anti-VEGF agents, with a statistically significant reduction in the number of intravitreal injections required compared with flexible regimens of aflibercept and ranibizumab.^16^

The reduction in the number of injections required for disease management reflects not only the benefit of faricimab in economic savings for the acquisition and administration costs but also how the treatment burden related to the diseases can be reduced. Previous studies have highlighted the impact of visits for intravenous injections on patient well-being, including pre-injection anxiety, discomfort, and pain, as well as the need for patients and caregivers to take time off work to attend appointments.^46–48^ This relates to the findings on the high economic burden of both diseases, associated with loss of productivity and work absenteeism of patients and caregivers in Colombia, which by 2022 represented a total of US $107 million.^10^

Our economic evaluation reflects how faricimab can reduce treatment burden, which is consistent with previous studies. The study by Li et al performed for the United Kingdom showed that faricimab allows the release of resources by requiring fewer injections, favoring its use in health systems with limited installed capacity.^49^ Similarly, Hurley et al found that faricimab represents a significant reduction in the treatment burden by improving the patient and caregiver experience in Canada.^50^ By reducing the treatment burden while maintaining health outcome gains and achieving savings in treatment-related costs, faricimab presents a valuable alternative for managing DME and nAMD from the patient’s perspective and for the Colombian health system.

Similarly, the cost-effectiveness analysis by Meunier et al, conducted from a societal perspective, showed that faricimab had a positive increase in societal welfare attributable to the reduction of inequalities in the treatment of DME. This result was measured with a composite outcome that assesses the degree of distribution of the health benefit among different socioeconomic groups, determining that the benefit is uniform across the entire population, suggesting that faricimab is both a cost-effective and equity-improving option for the treatment of DME.^51^

This study has some limitations that should be considered. The efficacy and treatment frequency reduction inputs were taken from the results of the available clinical evidence, including NMAs comparing faricimab with other anti-VEGF agents. The results of secondary studies may have limitations, as described in the NMAs by Watkins et al. and Samacá et al.^15,16^

It is also important to consider that the clinical studies that assessed the efficacy of faricimab in both DME and nAMD were noninferiority clinical trials, which may add uncertainty to the results by depending on the noninferiority margin selected.^52^ This uncertainty can be extended to the economic model; however, both the NMAs and the model itself use the absolute ETDRS letter gain reported in the studies, thus reducing the impact of assuming clinical equivalence between treatments based only on having achieved the endpoint of noninferiority in the clinical trials.

Another limitation might be that the utility values used in the economic model come from the study by Czoski-Murray et al^31^ and do not correspond to a preference assessment conducted in the Colombian population. To our knowledge, there is no study evaluating utility values for eye diseases in Colombia, limiting the use of local data for developing cost-effectiveness analyses.

Finally, a Markov model was adapted for this study, suitable for modeling chronic diseases. However, the model incorporates certain assumptions, including a 5-year treatment discontinuation rate and the rules for transitioning between health states based on VA levels. Similarly, the BIA contains assumptions, including the annual increase in market share and the proportion of prevalent cases with bilateral disease. Nonetheless, these parameters seek to reflect the progression of the disease under treatment and were validated during their construction^17^ and local adaptation with clinical experts; likewise, this model’s replication in several countries supports its internal and external consistency in the results.

## CONCLUSION

From the Colombian Health System perspective, considering a threshold of US $5988 in 2023 (86% of gross domestic product per capita), faricimab is a cost-effective alternative for both DME and nAMD, showing dominance over other anti-VEGF agents (aflibercept, ranibizumab, and brolucizumab) by achieving better health outcomes at a lower cost.

In addition, faricimab reduces the treatment burden and improves Colombian patients’ health-related quality of life by reducing the number of annual injections, with gains in QALYs compared with other treatments, freeing up healthcare resources. This allows the healthcare system to reallocate resources, improving healthcare efficiencies for the Colombian population.

### Disclosures

H.O., F.J.R., and C.S. have declared financial support from pharmaceutical companies, including Bayer, Novartis, Astellas, Abbvie, and Roche, for conferences and academic meetings, as well as for consulting fees and Advisory Boards. D.S.S., L.P.P., C.B. and M.H.Q. are employees of Roche. A.R. was an employee of Roche at the time of the study. M.H.Q. is a member of ACOFACLI (Asociación Colombiana de Farmacología Clínica). C.T. and D.R. are employees of IQVIA Colombia. IQVIA served as consultant company for Roche for the development of the study. None of the authors received any compensation for the authorship of this manuscript.

## Supplementary Material

Online Supplementary Material
