# Supplementary material for: Cost-Effectiveness and Budget Impact Analysis of the Use of Faricimab in Diabetic Macular Edema and Neovascular Age-Related Macular Degeneration in Colombia
Source: J Health Econ Outcomes Res. 2025 Mar 4;12(1):97–105. doi: 10.36469/001c.129832 (PMC11884430; doi:10.36469/001c.129832)
Supplement: Online Supplementary Material [file jheor_2025_12_1_129832_269524.pdf]

Cost-effectiveness and Budget Impact Analysis of the Use of Faricimab in Diabetic Macular Edema and Neovascular Age-Related Macular Degeneration in Colombia

Online Supplementary Material

Table S1. Administration, Supportive Care And Adverse Events Cost 2
Table S2. Distribution of Baseline Visual Acuity 3
Table S3. Utility Values According to Visual Acuity Score[4] - DME and nAMD 3
Table S4. Transition Probabilities at the End of Year 1 by Baseline Visual Acuity Status in Patients With DME 4
Table S5. Transition Probabilities During Year 2 in Patients With Diabetic Macular Edema 5
Table S6. Transition Probabilities During Year 3 and Onward in Patients With Diabetic Macular Edema 6
Table S7. Transition Probabilities at the End of Year 1 by Baseline Visual Acuity Status in Patients With Neovascular Age-Related Macular Degeneration 6
Table S8. Transition Probabilities During Year 2 in Patients With Neovascular Age-Related Macular Degeneration 7
Table S9. Transition Probabilities During Year 3 and Onwards in Patients With Neovascular Age-Related Macular Degeneration 7
Table S10. Probabilistic Sensitivity Analysis Parameters: Diabetic Macular Edema .. 8
Table S11. Probabilistic Sensitivity Analysis Parameters: Neovascular Age-Related Macular Degeneration 9

Figure S1. Tornado Diagram: Faricimab T&E vs Ranibizumab PRN for Net Monetary Benefit in Patients With Diabetic Macular Edema 10
Figure S2. Tornado Diagram*:* Faricimab T&E vs Aflibercept PRN for Net Monetary Benefit in Patients With Diabetic Macular Edema 10
Figure S3. Tornado Diagram: Faricimab T&E vs Brolucizumab 8-12 Weeks for Net Monetary Benefit in Patients With Diabetic Macular Edema 11
Figure S4. Tornado Diagram: Faricimab T&E vs Ranibizumab PRN for Net Monetary Benefit in Patients With Neovascular Age-Related Macular Degeneration 11
Figure S5. Tornado Diagram: Faricimab T&E vs Aflibercept T&E for Net Monetary Benefit in Patients With Neovascular Age-Related Macular Degeneration 11
Figure S6. Tornado Diagram: Faricimab T&E vs Brolucizumab 8-12 weeks for Net Monetary Benefit in Patients With Neovascular Age-Related Macular Degeneration 12

Table S1. Administration, Supportive Care And Adverse Events Cost

Cost Input	Usage in the Population (%)	Frequency	Unit Cost (US $)
**Administration costs**			
Administration of intravitreal injection	100	1 per administration	30.68
Optical coherence tomography	100	1 per administration	55.80
Consultant-led outpatient attendance	100	1 per administration	6.29
**Supportive care**[1]			
One-off visual impairment assessment	100	1 per patient	19.36
Uptake of low-vision aids	33	1 per year	170.81
Low vision rehabilitation	100	1 per year	38.73
Treatment of vision loss–related depression	39	1 per year	31.97
Hip replacement due to falls	5	1 per patient	3069.75
**Adverse Events**	**Probability per Cycle (nAMD)**	**Probability per Cycle (DME)**	**Cost per Event (US$)**
Cataracts	0.02	0.03	309.04
Dry eye	0	0.004	10.38
Endophthalmitis	0.02	0.02	608.27
Glaucoma	0	0.004	485.43
Keratouveitis	0	0.004	1.73
Intraocular pressure increased	0.01	0.004	157.96
Ocular ischemic syndrome	0	0.004	998.93
Retinal tear	0	0.01	784.10
Posterior capsule opacification	0	0.004	128.38
Rhegmatogenous retinal detachment	0.01	0.004	605.61
Uveitis	0.02	0.01	1.27
Viral uveitis	0.01	0.004	1.74
Vitreous hemorrhage	0.01	0.01	605.61

Abbreviations: DME, diabetic macular edema; nAMD: neovascular age-related macular degeneration.

Table S2. Distribution of Baseline Visual Acuity

Baseline Distribution	>85	85-71	70-56	55-41	40-26	≤25
Diabetic macular edema[2]						
First eye with DME at baseline	0.1	18.4	60.6	16.0	4.8	0.2
Second eye without EMD at baseline	0.1	20.0	63.9	12.3	3.6	0.0
Second eye with EMD at baseline	0.0	16.4	60.1	18.2	5.1	0.2
Neovascular age-related macular degeneration[3]						
First eye with nAMD at baseline	0.0	25.0	43.0	20.0	10.0	2.0
Second eye without nAMD at baseline	15.0	64.0	15.0	3.0	1.0	2.0
Second eye with nAMD at baseline	3.0	36.0	22.0	9.0	11.0	19.0

Abbreviations: DME, diabetic macular edema; nAMD, neovascular age-related macular degeneration.

Table S3. Utility Values According to Visual Acuity Score[4] - DME and nAMD

		Second Eye					
		>85	85-71	70-56	55-41	40-26	≤25
**First Eye**							
	>85	0.919	0.893	0.868	0.842	0.817	0.782
	85-71	0.893	0.808	0.783	0.758	0.732	0.697
	70-56	0.868	0.783	0.698	0.673	0.647	0.612
	55-41	0.842	0.758	0.673	0.588	0.562	0.527
	40-26	0.817	0.732	0.647	0.562	0.477	0.442
	≤25	0.782	0.697	0.612	0.527	0.442	0.326

Abbreviations: DME, diabetic macular edema; nAMD: neovascular age-related macular degeneration.

Table S4. Transition Probabilities at the End of Year 1 by Baseline Visual Acuity Status in Patients With DME

BCVA at Baseline	+2 VA HS	+1 VA HS	Stable	-1 VA HS	-2 VA HS
>85					
Faricimab	0.001	0.490	0.496	0.018	0.000
Aflibercept	0.000	0.346	0.632	0.012	0
Ranibizumab	0.000	0.182	0.774	0.044	0
Brolucizumab	0.000	0.440	0.548	0.007	0
85-71					
Faricimab	0.008	0.577	0.398	0.016	0.000
Aflibercept	0.003	0.438	0.546	0.013	0
Ranibizumab	0.000	0.241	0.701	0.048	0
Brolucizumab	0.006	0.531	0.451	0.007	0
70-56					
Faricimab	0.051	0.638	0.302	0.015	0.000
Aflibercept	0.021	0.521	0.444	0.013	0
Ranibizumab	0.005	0.316	0.629	0.053	0
Brolucizumab	0.041	0.598	0.348	0.007	0
55-41					
Faricimab	0.252	0.549	0.181	0.011	0.000
Aflibercept	0.130	0.540	0.318	0.011	0
Ranibizumab	0.038	0.385	0.523	0.054	0
Brolucizumab	0.220	0.554	0.225	0.006	0
40-26					
Faricimab	0.678	0.252	0.061	0.005	0.000
Aflibercept	0.488	0.359	0.151	0.007	0
Ranibizumab	0.225	0.375	0.357	0.046	0
Brolucizumab	0.635	0.277	0.080	0.002	0

Abbreviations: BCVA, best-corrected visual acuity; DME, diabetic macular edema; HS, health state; VA, visual acuity.

Table S5. Transition Probabilities During Year 2 in Patients With Diabetic Macular Edema

	+1 Visual Acuity Health State	Stable	-1 Visual Acuity Health State
Faricimab	0.000	0.973	0.027
Aflibercept	0.000	0.973	0.027
Ranibizumab	0.000	0.973	0.027
Brolucizumab	0.000	0.973	0.027

Table S6. Transition Probabilities During Year 3 and Onward in Patients With Diabetic Macular Edema

	Stable	-1 Visual Acuity Health State	-2 Visual Acuity Health State
Faricimab	0.931	0.069	0.000
Aflibercept	0.931	0.069	0.000
Ranibizumab	0.931	0.069	0.000
Brolucizumab	0.931	0.069	0.000

Table S7. Transition Probabilities at the End of Year 1 by Baseline Visual Acuity Status in Patients With Neovascular Age-Related Macular Degeneration

BCVA at Baseline	+2 VA HS	+1 VA HS	Stable	-1 VA HS	-2 VA HS
>85					
Faricimab	0.002	0.311	0.607	0.048	0.031
Aflibercept	0.001	0.195	0.707	0.062	0.032
Ranibizumab	0.003	0.248	0.677	0.050	0.024
Brolucizumab	0.001	0.191	0.715	0.064	0.032
**85-71**					
Faricimab	0.009	0.380	0.521	0.052	0.030
Aflibercept	0.006	0.251	0.643	0.073	0.032
Ranibizumab	0.009	0.311	0.599	0.056	0.024
Brolucizumab	0.006	0.245	0.650	0.075	0.033
70-56					
Faricimab	0.490	0.704	0.821	0.515	0.858
Aflibercept	0.025	0.306	0.553	0.078	0.029
Ranibizumab	0.037	0.371	0.506	0.061	0.021
Brolucizumab	0.025	0.299	0.562	0.082	0.031
55-41					
Faricimab	0.121	0.471	0.328	0.055	0.027
Aflibercept	0.094	0.349	0.446	0.081	0.026
Ranibizumab	0.132	0.403	0.390	0.060	0.018
Brolucizumab	0.093	0.343	0.454	0.084	0.026
40-26					
Faricimab	0.330	0.399	0.205	0.048	0.022
Aflibercept	0.288	0.324	0.299	0.070	0.019
Ranibizumab	0.363	0.340	0.237	0.047	0.013
Brolucizumab	0.280	0.316	0.304	0.072	0.020

Abbreviations: BCVA, best-corrected visual acuity; HS, health state; VA, visual acuity.

Table S8. Transition Probabilities During Year 2 in Patients With Neovascular Age-Related Macular Degeneration

	+1 Visual Acuity Health State	Stable	-1 Visual Acuity Health State
Faricimab	0.000	0.948	0.052
Aflibercept	0.000	0.948	0.052
Ranibizumab	0.000	0.948	0.052
Brolucizumab	0.000	0.948	0.052

Table S9. Transition Probabilities During Year 3 and Onwards in Patients With Neovascular Age-Related Macular Degeneration

	Stable	-1 Visual Acuity Health State	-2 Visual Acuity Health State
Faricimab	0.895	0.105	0.000
Aflibercept	0.895	0.105	0.000
Ranibizumab	0.895	0.105	0.000
Brolucizumab	0.895	0.105	0.000

Table S10. Probabilistic Sensitivity Analysis Parameters: Diabetic Macular Edema

**Cost-Effectiveness Model (PSA): DME**		
**Variable**	**Distribution**	**Mean (SD)**
Average age of cohorts (years)	Normal	54.5 (2.73)
First eye at baseline: Distribution of mean BCVA	Normal	61.85 (3.09)
Second eye without DME at baseline: Distribution of mean BCVA	Normal	63.13 (63.13)
Second eye with DME at baseline: Distribution of mean BCVA	Normal	61.11 (61.11)
Incidence of DME in 2nd eye at baseline	Normal	47 (2)
Monthly incidence of DME in 2nd eye	Normal	1 (0.1)
Annual mean BCVA change beyond 2 years on treatment	Normal	-0.41 (0.09)
Consultant led outpatient attendance (cost)	Gamma	6.29 (0.31)
OCT (cost)	Gamma	55.8 (2.79)
Administration of injection (cost)	Gamma	30.68 (1.53)
Administration and monitoring visits assigned to the 2nd eye (If both eyes are treated)	Beta	100 (0.05)
One-off visual impairment assessment (cost)	Gamma	19.36 (0.97)
Uptake of low vision aids (cost)	Gamma	170.81 (8.54)
Low vision rehabilitation (cost)	Gamma	38.73 (1.94)
Treatment of vision loss–related depression (cost)	Gamma	31.97 (1.6)
Hip replacement due to falls (cost)	Gamma	3069.75 (153.49)
Uptake of low vision aids (proportion)	Beta	33 (2)
Hip replacement due to falls (proportion)	Beta	5 (0.3)
Cataracts (cost)	Gamma	309.04 (15.45)
Endophthalmitis (cost)	Gamma	608.27 (30.41)
Dry eye (cost)	Gamma	10.38 (0.52)
Glaucoma (cost)	Gamma	485.43 (24.27)
Keratouveitis (cost)	Gamma	1.73 (0.09)
Intraocular pressure increased (cost)	Gamma	157.96 (7.9)
Ocular ischemic syndrome (cost)	Gamma	128.38 (6.42)
Retinal tear (cost)	Gamma	784.1 (39.21)
Posterior capsule opacification (cost)	Gamma	128.38 (6.42)
Rhegmatogenous retinal detachment (cost)	Gamma	605.61 (30.28)
Uveitis (cost)	Gamma	1.27 (0.06)
Viral uveitis (cost)	Gamma	1.74 (0.09)
Vitreous hemorrhage (cost)	Gamma	605.61 (30.28)
Utility decrements for administration	Normal	0.02 (0.00)
Utility by VA state (regression – constant)	Beta	0.86 (0.07)
Utility by VA state (regression – standard error)	Normal	-0.37 (0.05)
Utility by VA state (regression – weighted standard error)	Normal	0.23 (0.00)

Abbreviations: BCVA, best corrected visual acuity; DME, diabetic macular edema; OCT, optical coherence tomography; PSA, probabilistic sensitivity analysis; VA, visual acuity.

Table S11. Probabilistic Sensitivity Analysis Parameters: Neovascular Age-Related Macular Degeneration

Cost-Effectiveness Model (PSA) – nAMD		
**Variable**	**Distribution**	**Mean (SD)**
Average age of cohorts (years)	Normal	74.0 (3.70)
First eye at baseline: Distribution of mean BCVA	Normal	59.74 (2.99)
Second eye without DME at baseline: Distribution of mean BCVA	Normal	75.34 (3.77)
Second eye with DME at baseline: Distribution of mean BCVA	Normal	55.06 (2.75)
Incidence of DME in 2nd eye at baseline	Normal	7.3 (0.4)
Monthly incidence of DME in 2nd eye	Normal	1.4 (0.1)
Annual mean BCVA change beyond 2 years on treatment	Normal	-2.50 (0.13)
Consultant led outpatient attendance (cost)	Gamma	6.29 (0.31)
OCT (cost)	Gamma	55.8 (2.79)
Administration of injection (cost)	Gamma	30.68 (1.53)
Administration and monitoring visits assigned to the 2nd eye (If both eyes are treated)	Beta	100 (0.05)
One-off visual impairment assessment (cost)	Gamma	19.36 (0.97)
Uptake of low-vision aids (cost)	Gamma	170.81 (8.54)
Low vision rehabilitation (cost)	Gamma	38.73 (1.94)
Treatment of vision loss–related depression (cost)	Gamma	31.97 (1.6)
Hip replacement due to falls (cost)	Gamma	3069.75 (153.49)
Uptake of low vision aids (proportion)	Beta	33 (2)
Hip replacement due to falls (proportion)	Beta	5 (0.3)
Cataracts (cost)	Gamma	309.04 (15.45)
Endophthalmitis (cost)	Gamma	608.27 (30.41)
Intraocular pressure increased (cost)	Gamma	157.96 (7.9)
Rhegmatogenous retinal detachment (cost)	Gamma	605.61 (30.28)
Uveitis (cost)	Gamma	1.27 (0.06)
Viral uveitis (cost)	Gamma	1.74 (0.09)
Vitreous haemorrhage (cost)	Gamma	605.61 (30.28)
Utility decrements for administration	Normal	0.02 (0.00)
Utility by VA state (regression – constant)	Beta	0.86 (0.07)
Utility by VA state (regression – standard error)	Normal	-0.37 (0.05)
Utility by VA state (regression – weighted standard error)	Normal	0.23 (0.00)

Abbreviations: BCVA, best corrected visual acuity; nAMD: neovascular age-related macular degeneration; OCT, optical coherence tomography; PSA, probabilistic sensitivity analysis; VA, visual acuity.

Figure S1. Tornado Diagram: Faricimab T&E vs Ranibizumab PRN for Net Monetary Benefit in Patients With Diabetic Macular Edema

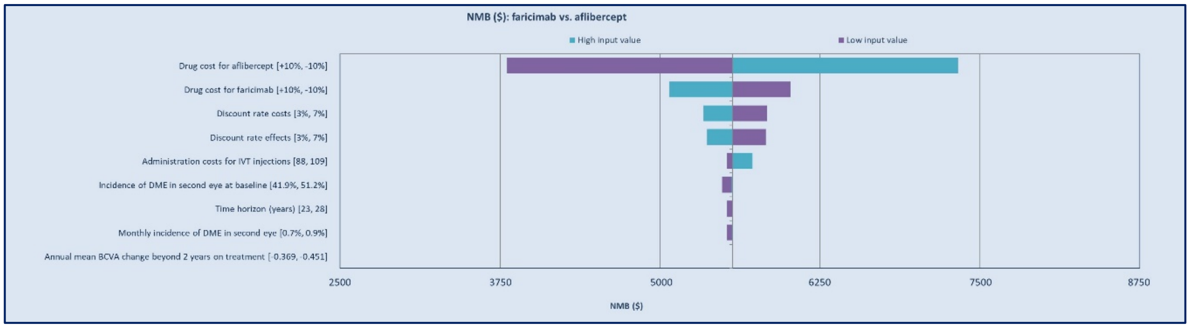

Abbreviations: DME, diabetic macular edema; NMB, net monetary benefit; PRN, as needed; T&E, treat and extend.

Note: US $1 = Col$4325.

Figure S2. Tornado Diagram*:* Faricimab T&E vs Aflibercept PRN for Net Monetary Benefit in Patients With Diabetic Macular Edema

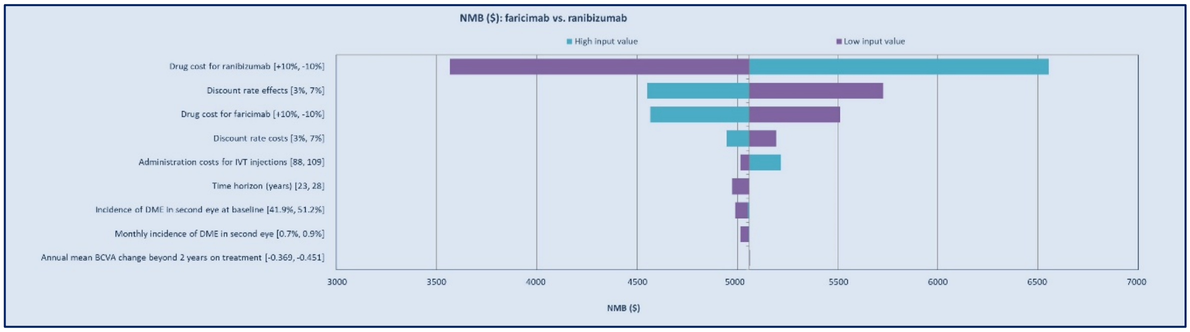

Abbreviations: DME, diabetic macular edema; NMB, net monetary benefit; PRN, as needed; T&E, treat and extend.

Note: US $1 = Col$4325.

Figure S3. Tornado Diagram: Faricimab T&E vs Brolucizumab 8-12 Weeks for Net Monetary Benefit in Patients With Diabetic Macular Edema

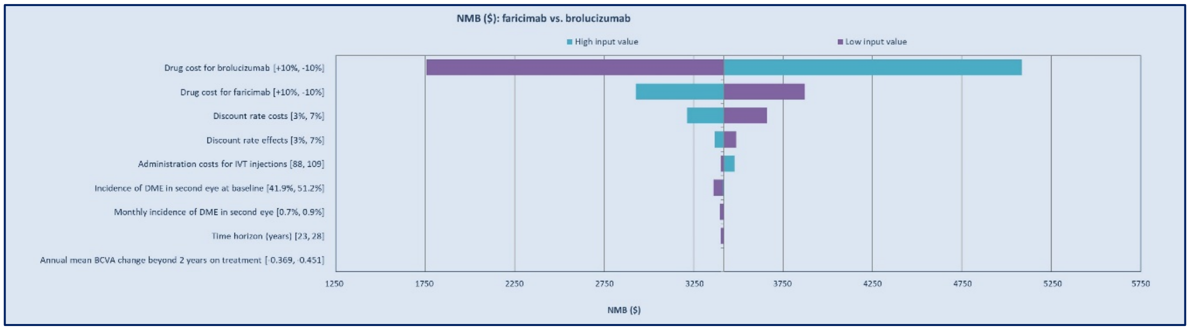

Abbreviations: DME, diabetic macular edema; NMB, net monetary benefit; PRN, as needed; T&E, treat and extend.

Note: US $1 = Col$4325.

Figure S4. Tornado Diagram: Faricimab T&E vs Ranibizumab PRN for Net Monetary Benefit in Patients With Neovascular Age-Related Macular Degeneration

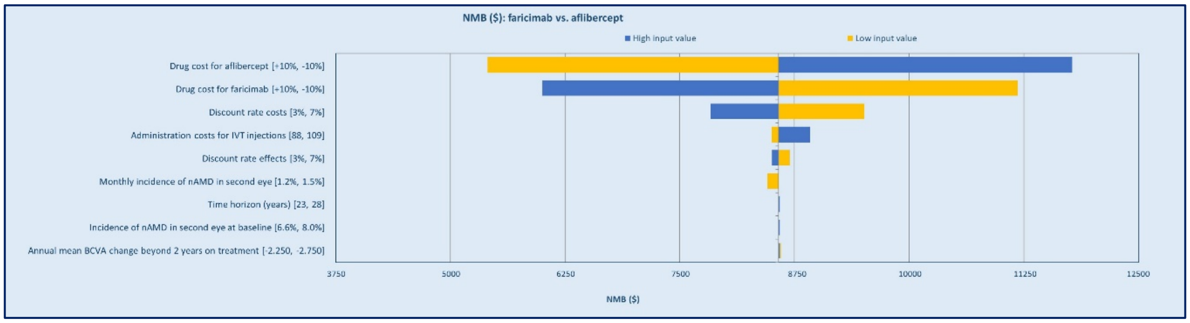

Abbreviations: nAMD, neovascular age-related macular degeneration; NMB, net monetary benefit; T&E: treat and extend.

Note: US $1 = Col$4325.

Figure S5. Tornado Diagram: Faricimab T&E vs Aflibercept T&E for Net Monetary Benefit in Patients With Neovascular Age-Related Macular Degeneration

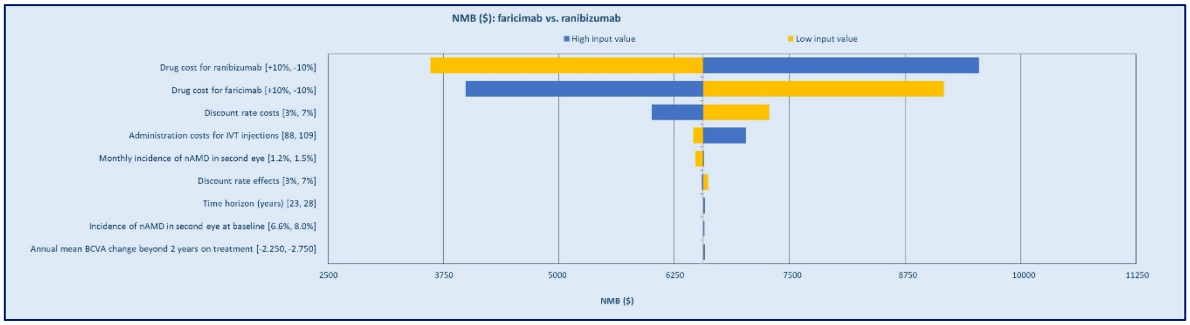

Abbreviations: nAMD, neovascular age-related macular degeneration; NMB, net monetary benefit; T&E, treat and extend.

Note: US $1 = Col$4325.

Figure S6. Tornado Diagram: Faricimab T&E vs Brolucizumab 8-12 weeks for Net Monetary Benefit in Patients With Neovascular Age-Related Macular Degeneration

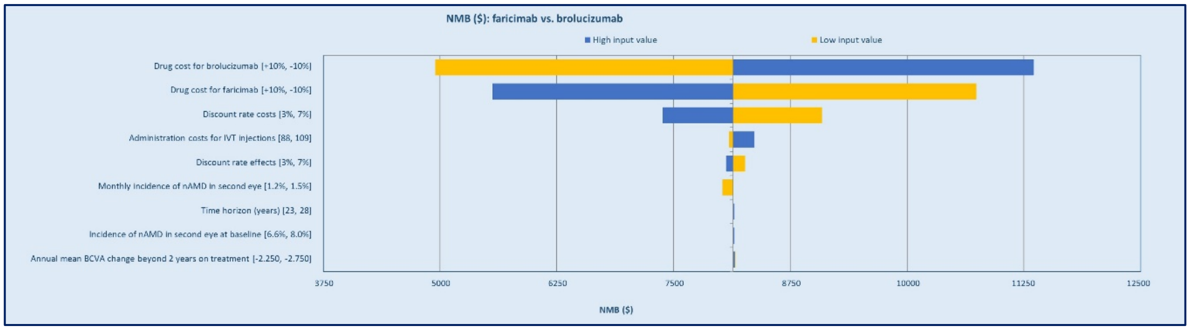

Abbreviations: nAMD, neovascular age-related macular degeneration; NMB, net monetary benefit; T&E, treat and extend.

Note: US $1 = Col$4325.

References

1. Meads C, Hyde C. What is the cost of blindness? *Br J Ophthalmol*. 2003;87(10):1201-1204.
2. Wykoff CC, Abreu F, Adamis AP, et al. Efficacy, durability, and safety of intravitreal faricimab with extended dosing up to every 16 weeks in patients with diabetic macular oedema (YOSEMITE and RHINE): two randomised, double-masked, phase 3 trials. *Lancet*. 2022;399(10326):741-755.
3. National Institute for Health and Care Excellence. *NICE. NG82 Macular degeneration. Appendix J: Health Econ*. 2018. https://www.nice.org.uk/guidance/ng82/evidence/appendix-j-health-economics-pdf-170036251093
4. Czoski-Murray C, Carlton J, Brazier J, Young T, Papo NL, Kang HK. Valuing condition-specific health states using simulation contact lenses. *Value Health*. 2009;12(5):793-799.
